# Computational Investigations on the Binding Mode of Ligands for the Cannabinoid-Activated G Protein-Coupled Receptor GPR18

**DOI:** 10.3390/biom10050686

**Published:** 2020-04-29

**Authors:** Alexander Neumann, Viktor Engel, Andhika B. Mahardhika, Clara T. Schoeder, Vigneshwaran Namasivayam, Katarzyna Kieć-Kononowicz, Christa E. Müller

**Affiliations:** 1 PharmaCenter Bonn, Pharmaceutical Institute, Pharmaceutical Sciences Bonn (PSB), Pharmaceutical & Medicinal Chemistry, University of Bonn, 53121 Bonn, Germany; 2 Research Training Group 1873, University of Bonn, 53127 Bonn, Germany; 3 Department of Technology and Biotechnology of Drugs, Faculty of Pharmacy, Jagiellonian University Medical College, Medyczna 9, 30-688 Krakow, Poland

**Keywords:** cannabinoid, docking studies, GPCR, GPR18, MD simulation, orphan GPCRs

## Abstract

GPR18 is an orphan G protein-coupled receptor (GPCR) expressed in cells of the immune system. It is activated by the cannabinoid receptor (CB) agonist ∆^9^-tetrahydrocannabinol (THC). Several further lipids have been proposed to act as GPR18 agonists, but these results still require unambiguous confirmation. In the present study, we constructed a homology model of the human GPR18 based on an ensemble of three GPCR crystal structures to investigate the binding modes of the agonist THC and the recently reported antagonists which feature an imidazothiazinone core to which a (substituted) phenyl ring is connected via a lipophilic linker. Docking and molecular dynamics simulation studies were performed. As a result, a hydrophobic binding pocket is predicted to accommodate the imidazothiazinone core, while the terminal phenyl ring projects towards an aromatic pocket. Hydrophobic interaction of Cys251 with substituents on the phenyl ring could explain the high potency of the most potent derivatives. Molecular dynamics simulation studies suggest that the binding of imidazothiazinone antagonists stabilizes transmembrane regions TM1, TM6 and TM7 of the receptor through a salt bridge between Asp118 and Lys133. The agonist THC is presumed to bind differently to GPR18 than to the distantly related CB receptors. This study provides insights into the binding mode of GPR18 agonists and antagonists which will facilitate future drug design for this promising potential drug target.

## 1. Introduction

G protein-coupled receptors (GPCR) represent the largest family of membrane proteins in eukaryotes. They are structurally characterized by seven transmembrane (TM) regions connected by three extracellular (ECL1-3) and three intracellular loops (ICL1-3), an extracellular N-terminal and an intracellular C-terminal domain. Upon binding of the cognate agonist (e.g., biogenic amine neurotransmitter, nucleotide, lipid, amino acid, peptide, glycoprotein) conformational changes are induced. These result in coupling with G proteins, and thereby transducing information from the extracellular to the intracellular compartment and inducing or inhibiting downstream signaling pathways [1,2]. Despite persistent efforts, nearly 100 GPCRs remain orphan, with their endogenous ligands unidentified or unconfirmed [3]. The functionalities and roles of orphan GPCRs under (patho)physiological conditions are in most cases poorly understood. The identification of the endogenous ligands would be helpful for target validation studies and the design of novel therapeutic drugs for orphan GPCRs.

GPR18 is such an orphan GPCR of therapeutic interest, phylogenetically belonging to the δ-branch of class A, rhodopsin-like GPCRs. GPR18 was first described in 1997 and reported to be highly expressed in different tissues and cell lines of the immune system, including spleen, thymus, and leukocytes [4]. The role of GPR18 is still unclear and controversially debated. GPR18 has been proposed by independent groups to be involved in immunological [5,6,7,8] and neurodegenerative processes including Alzheimer’s disease and multiple sclerosis [9,10,11,12,13]. Based on the observation that the activation of GPR18 lowers the intraocular pressure in mice, GPR18 agonists have been proposed for the treatment of glaucoma [14,15]. Antagonists targeting GPR18 may be effective as anticancer drugs [16,17,18], since the receptor was found to be abundantly overexpressed in melanoma metastases and reported to contribute to tumor cell survival through inhibition of apoptosis [17].

**Figure 1:**
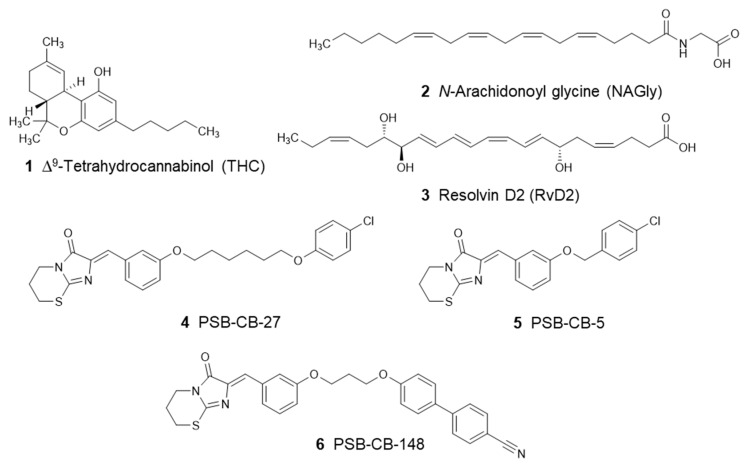
Structures of proposed GPR18 agonists (**1**–**3**) and antagonists (**4**–**6**).

In recent years, several studies aimed at the deorphanization of GPR18 have been published. Due to the lack of selective agonists, the moderately potent cannabinoid (CB) receptor agonist ∆^9^-tetrahydrocannabinol (THC, **1**) has been used in pharmacological studies to activate human GPR18, which led to the suggestion to classify GPR18 as a cannabinoid receptor subtype besides CB_1_ and CB_2_ [19,20,21,22]. *N*-Arachidonoylglycine (NAGly, **2**) and resolvin D2 (RvD2, **3**) were proposed as endogenous agonists of GPR18 [23,24]. However, independent confirmation for both lipids is still lacking, as other groups, including ours, have not been able to confirm their activation of GPR18 [25,26]. We recently described the first GPR18 antagonists based on an imidazothiazinone core structure [21,27]. These were discovered by screening a compound library at the human receptor in a β-arrestin recruitment assay using enzyme complementation technology and THC as an agonist. Based on the screening results, a library of imidazothiazinones was synthesized and their structure–activity relationships (SARs) were investigated. PSB-CB-27 (**4**) and PSB-CB-5 (**5**; for structures, see Figure 1) were reported as the first potent and selective GPR18 antagonists [21].

In the present study, we constructed a homology model of the human GPR18 to elucidate the binding mode of the only confirmed agonist so far, the natural product THC, and of selected antagonists by docking and molecular dynamics (MD) simulation studies. Insights into the binding interactions of agonists and antagonists will provide a basis for the rational design of more potent ligands and may eventually contribute to the deorphanization of GPR18.

## 2. Material and Methods

### 2.1. Homology Modeling

The crystal structures of the murine μ-opioid receptor in complex with the agonist BU72 (PDB-ID: 5C1M), the human P2Y_1_ receptor in complex with the allosteric antagonist BPTU (PDB-ID: 4XNV) and the zebrafish lysophosphatidic acid receptor LPA6 in complex with 1-oleoyl-*R*-glycerol (PDB-ID: 5XSZ) were obtained from the Research Collaboratory for Structural Bioinformatics (RCSB) Protein Data Bank (PDB) [28,29,30]. The crystal structures of all three receptors were used as templates for generating homology models of the human GPR18 sequence (accession number: Q14330) retrieved from the UniProt sequence database (http://www.uniprot.org) [31]. The sequences of the murine μ-opioid receptor, the P2Y_1_R, and the zebrafish lysophosphatidic acid receptor LPA6 were aligned with that of the human GPR18 using Clustal Omega [32]. We generated 500 models for the human GPR18 based on the triple template approach using the standard comparative modeling automodel class implemented in MODELLER (version 9.16, University of California, San Francisco, CA, USA). To ensure correct tertiary protein structure prediction, we introduced a disulfide bridge between Cys94 and Cys172. The best model was selected on the basis of Discrete Optimized Protein Energy (DOPE) scores calculated for the models [33,34]. The generated models were analyzed, and the best models for the human GPR18 were used to perform molecular docking studies, based on the DOPE and GA341 score, PROSA II Z score, and Ramachandran plots. We took into account that the X-ray crystal structure of the lysophosphatidic acid receptor LPA6 is missing part of the ECL2, likely due to low resolution and high flexibility of that region. Nevertheless, we decided to include LPA6 as a template for model generation as it might provide valuable information, e.g., regarding the transmembrane domains and the ligand-binding site. Sequences for the cannabinoid receptors CB_1_ (P21554) and CB_2_ (P34972) were retrieved from UniProt.

### 2.2. Docking Studies

Prior to docking, the homology model of the human GPR18 was prepared using the Protein Preparation Wizard module implemented in Schrödinger [34,35]. In the first step for protein preparation, we preprocessed the structure using the standard protocol at pH 7.4. Docking was performed using Induced Fit Docking (IFD) and Glide as implemented in Schrödinger release 2016 [35,36,37]. In the first step of IFD, Glide ligand docking was performed by removing the side chains of the amino acids in the selected binding pocket. In the second phase of docking, Prime was applied to refine the nearby residues and to optimize the side chains. In the final docking phase, the ligand was re-docked into all induced fit protein structures that were within 30 kcal/mol of the lowest energy structure, by using the Glide XP scoring function. A receptor grid center was specified on the basis of preliminary docking studies, resulting in the highest docking scores for the centroid of Lys174 with a cubic grid side length of 10 Å. Preliminary ensemble docking studies provided highest docking scores and consistent SARs explanation for this selection as well as comparison with published cannabinoid receptor X-ray crystal structures [38].

During the docking simulations, the receptor and the ligands were kept flexible. Following docking, the resulting poses of the best model were selected using the IFD scores and Prime Energy as representative values. The conformations of the docked ligands within an energy window of 2.5 kcal/mol were considered. For Glide docking, the following standard parameters were selected: receptor van der Waals scaling, 0.50, ligand van der Waals scaling, 0.50, and a maximum of 20 poses per ligand. Residues within 5.0 Å of the ligand poses were refined, and the side chains were optimized. The best docking pose was selected based on the IFD score and Prime Energy values.

### 2.3. Compounds

Synthesis of the compounds which are utilized in this computational study as performed in the Department of Technology and Biotechnology of Drugs Jagiellonian University at Kraków, Poland, and potencies of the compounds were determined at the Department of Pharmaceutical & Medicinal Chemistry, Pharmaceutical Institute, University of Bonn, Germany, as previously reported [21,27]. The synthesis and biological evaluation of the new potent GPR18 antagonist 6 will be published elsewhere.

### 2.4. Molecular Dynamics Simulation

We selected several successful MD simulations as starting points for our runs [39,40,41,42]. The GPR18 complexes and the unbound GPR18 structure were prepared using the method described above to determine the protein protonation state at pH 7.4. The obtained structures were processed to the CHARMM-GUI molecular simulation program [43,44,45]. The forcefield CHARMM36m was applied for all simulation runs. Ligand parameters were obtained separately from Schrödinger. The orientation of the protein in the phosphatidylcholine lipid bilayer (POPC) was determined using the orientation of proteins in membranes (OPM) database [46]. The cubic water box size was adjusted to the structure size of 20 Å and filled with 0.15 M KCl solution. Water molecules were treated with the transferable intermolecular potential with a 3 points (TIP3P) water model [47]. Equilibration steps for all structures were divided into six steps using NAMD2 [48]. For the first three steps, we selected a runtime of 250 ps in 1 fs intervals. For the last three steps, we selected an equilibration runtime of 2 ns in 2 fs intervals. The system was heated from 0 to 303.15 K during equilibration using the NPT ensemble. During production stages, the system was kept at 303.15 K. Temperature was regulated using the Langevin dynamics thermostat. Production runs were performed for 4 × 50 ns with 4 fs intervals (eventually amounting to 200 ns), and frames were collected every 40 ps using ACEMD by Acellera^®^ with the NVT ensemble [49].

## 3. Results

So far, no X-ray crystal structure of GPR18 has been published. After performing a BLAST search, three crystal structures with highest sequence identity and overall sequence coverage were chosen as templates: the murine μ-opioid receptor (PDB-ID: 5C1M) in complex with an agonist, the human P2Y_1_R (PDB-ID: 4XNV) in complex with an allosteric antagonist, and the zebrafish lysophosphatidic acid receptor LPA6 (PDB-ID: 5XSZ) in complex with oleoyl-*R*-glycerol, showing sequence identities of 24.8%, 25.5% and 27.3%, respectively [28,29,30,50]. Multiple template approaches had been reported to compensate for poor sequence similarity for receptors lacking a template with sequence similarity above 30% [51,52]. Therefore, we decided to include all three templates into the process of homology modeling, although they represent different states of receptor activation. Structures of class A GPCRs belonging to the same δ-branch as GPR18 (P2Y_1_, LPA6) andone GPCR that is activated by a lipid like GPR18 (LPA6) were selected. BBy this approach, we expected to compensate for gaps and mismatching residues which would be present in a single template approach. The multiple sequence alignment is shown in Figure S1 of Supporting Information.

We subsequently investigated the binding modes of imidazothiazinone antagonists and of the agonist THC in the homology model of the human GPR18. To this end, we used the Induced Fit module implemented in Maestro Schrödinger to propose a binding mode for the selected ligands and to rationalize the potency values obtained in biological studies. Imidazothiazinone derivatives **4** and **5** were selected as representative potent antagonist structures. In addition to the imidazothiazinone core, they both possess a 4-chlorophenoxy substituent connected by a linker which differs in length. For the investigated antagonists **4** and **5**, IC_50_ values of 0.650 and 0.279 µM had been determined [21,27]. In order to investigate whether the proposed antagonist–GPR18 complexes are stable, we performed a 200 ns MD simulation study. Furthermore, we rationalized the SARs of related GPR18 antagonists using a structure-based approach.

### 3.1. Docking Studies of Antagonists

Recently, several studies on molecular modeling of the human GPR18 have been published [53,54,55,56,57]. However, neither binding mode predictions of THC (or other agonists) nor MD simulations of ≥200 ns of antagonist–GPR18 complexes have been published so far. Kuder et al. reported molecular modeling and docking studies, creating a homology model of the human GPR18 based on the crystal structure of the antagonist-bound human P2Y_1_ receptor [53]. The imidazothiazinone group of antagonist **5** was predicted to point into a deeper binding pocket towards TM3 where it was hypothesized to form a hydrogen bond with Arg191^5.42^. The results obtained in the present study indicate a different binding mode which is supported by comprehensive SAR data and based on an ensemble of templates for homology model generation rather than a single, low homology template as in the previous study [53].

**Figure 2:**
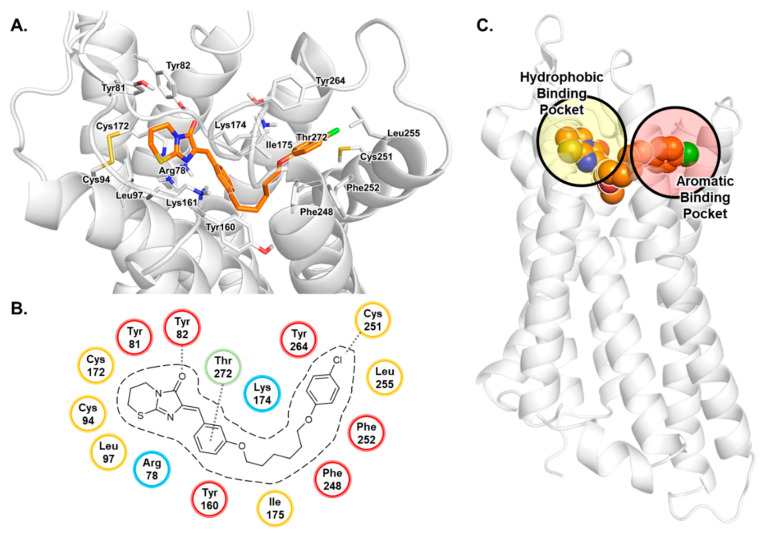
Proposed binding mode of antagonist **4**. (**A**) Docked pose of **4** in complex with the homology model of the human GPR18 shown with the residues forming the binding pocket. The receptor is displayed in cartoon representation, the amino acid residues (white) and compound **4** (orange) are shown as stick models. Oxygen atoms are colored in red, nitrogen atoms in blue, chlorine in green and sulfur atoms in yellow. (**B**) Schematic 2D representation of the binding pocket. Lipophilic amino acids are colored in yellow, hydrophilic ones in blue, aromatic ones in red, amino acid residues with mixed properties in green. (**C**) Schematic presentation of the homology model of GPR18 in complex with antagonist **4**. The imidazothiazinone moiety is predicted to bind in the hydrophobic binding pocket consisting of residues of TM3 and ECL2. The 4-chlorophenyl moiety binds in the aromatic binding pocket consisting of residues of TM6 and TM7. Cys251^6.54^ in the aromatic binding pocket most likely interacts with hydrophobic substituents in position 4 of the phenoxy (**4**) or benzyloxy (**5**) moiety of the antagonists.

**Figure 3:**
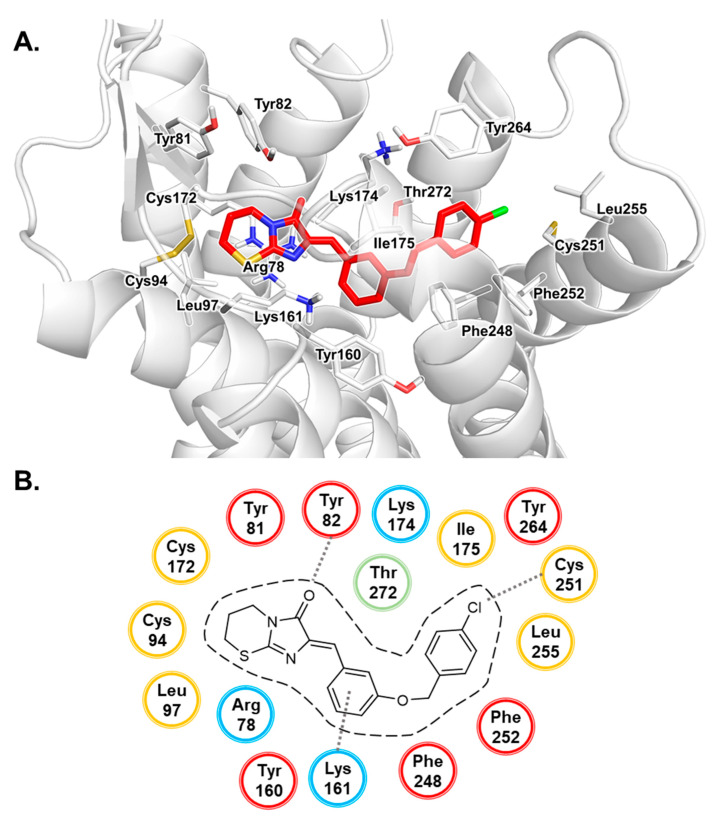
Proposed binding mode of antagonist **5**. (**A**) Docked pose of **5** in complex with the homology model of GPR18 shown with the residues forming the binding pocket. (**B**) Schematic 2D representation of the binding pocket. For color code, see Figure 2.

The proposed binding mode for antagonist **4** based on the performed docking studies is presented in Figure 2. Antagonist **4** is predicted to bind in the upper third part of the receptor, extending from a hydrophobic cavity formed by ECL2, TM2 and TM3 to an aromatic binding pocket formed by TM6 and TM7, which is a common motif for several GPCRs [58,59,60,61]. Compound **4** likely binds with its imidazothiazinone moiety close to the conserved disulfide bridge of Cys94^3.25^ and Cys172^ECL2^. Both cysteines and Leu97^3.28^ form a lipophilic binding pocket which is predicted to accommodate the thiazine ring. The keto group of the imidazolone ring likely forms an H-bond with Tyr82^2.64^. Due to the close proximity of Arg78^2.60^, cation–π interactions with the imidazolone system are feasible. The benzylidene ring may extend towards the center of the receptor, where hydrophobic interactions with Thr272^7.39^ are possible. The hexyloxy linker could bind with several hydrophobic residues (Tyr160^4.64^, Ile175^ECL2^, Phe248^6.51^, Met275^7.42^) towards an aromatic binding pocket formed by side chains of TM6 and TM7. Additional van der Waals forces for hydrophobic interactions with the benzylidene moiety and the hexyloxy linker may be provided by the alkyl chain of Lys174^ECL2^. Several aromatic (Phe248^6.51^, Phe252^6.55^, Tyr264^7.31^) and hydrophobic (Cys251^6.54^ and Leu255^6.58^) residues of TM6 and TM7 are predicted to form the binding pocket accommodating the 4-chlorophenoxy moiety of compound **5** (see Figure 3).

The smaller antagonist **5** can occupy the same binding cavity as antagonist **4** (see Figure 3). The imidazothiazinone moiety of both compounds can reach the same binding pose. Due to the missing linker, the benzylidine ring is predicted to exhibit an upward shift towards ECL2 where additional cation–π interactions with Lys161^ECL2^ can be realized. In both cases, the chlorine atoms on the terminal phenyl ring can reach the same binding cavity consisting of aromatic and hydrophobic residues of TM6 and TM7 close to Cys251^6.54^. Therefore, we expect halogen or methyl substitutions to interact analogously with Cys251^6.54^. These findings suggest that hydrophobic substituents in position 4 of the terminal phenyl ring of the antagonists are necessary for proper hydrophobic interaction with Cys251^6.54^ resulting in increased potency.

### 3.2. MD Simulation Study of Antagonists

**Figure 4:**
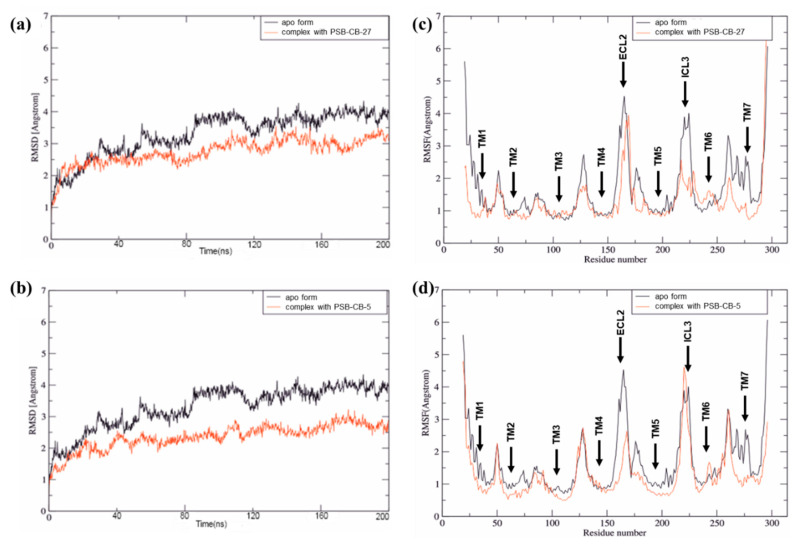
(**a**,**b**) Root mean square deviation (RMSD) curves for the 200 ns MD simulation runs of the GPR18 complex with antagonist **4** (**a**) and antagonist **5** (**b**). (**c**,**d**) Root mean square fluctuation (RMSF) curves of the molecular dynamics (MD) simulation for complexes with antagonist **4** (**c**) and **5** (**d**). Curves of the complexes are colored in orange, and the curve of the apo form of the receptor in black.

Both antagonist–GPR18 complexes were stable during the 200 ns MD simulation runs, which supports our prediction of the binding pocket based on docking studies. The duration of the MD simulation runs was in accordance with similar studies performed for other GPCRs [62,63,64,65]. The behavior of antagonists **4** and **5** in the homology model of GPR18 during the 200 ns MD simulation is presented from a bird’s eye view perspective in Supplementary Information Figures S2 and S3. The 0 ns state refers to the structure of the docked complex after equilibration. The course of the root mean square deviation (RMSD) indicates that the complex of GPR18 with antagonist **5** reached an equilibrated state after approximately 50 ns, and after approximately 100 ns for antagonist **4** (see Figure 4). Compared to the unbound GPR18 structure, the complex of GPR18 with antagonist **5** showed decreased root mean square fluctuation (RMSF) values for TM1, TM2, TM3, TM5 and TM7, and for ECL2 and ECL3, indicating stabilization of these regions upon antagonist binding. Similar results were observed for the complex with the larger antagonist **4**, where decreased RMSF values were observed for TM7, ECL2 and ECL3, and ICL2 and ICL3 when compared to the unbound structure. The concept of stabilization of an inactive conformation of the target GPCR upon antagonist binding was postulated for several receptors and supported by mutagenesis experiments, biophysical studies and MD simulations [58,66,67,68]. This had also been observed for the P2Y_1_ receptor which belongs to the same δ-branch of the class A family of GPCRs. The P2Y_1_ receptor can be blocked by structurally distinct antagonists that bind to different binding sites, the nucleotide analog MRS2500 and the urea derivative BPTU—both of which stabilize an ionic lock between an aspartic acid residue of ECL2 and an arginine of TM7 [69]. During MD simulations for 2 µs, RSMD values had been significantly lower for the complexes with an antagonist as compared to those with the P2Y_1_ receptor agonist ADP [69]. A shift in TM3, TM6 and TM7 in the simulation runs with the agonists created a void resulting in receptor activation through a bulk water influx into the binding pocket [69]. Similar observations were reported for several class A family members of GPCRs including µ-opioid receptors and adenosine receptors [70,71,72,73].

**Figure 5:**
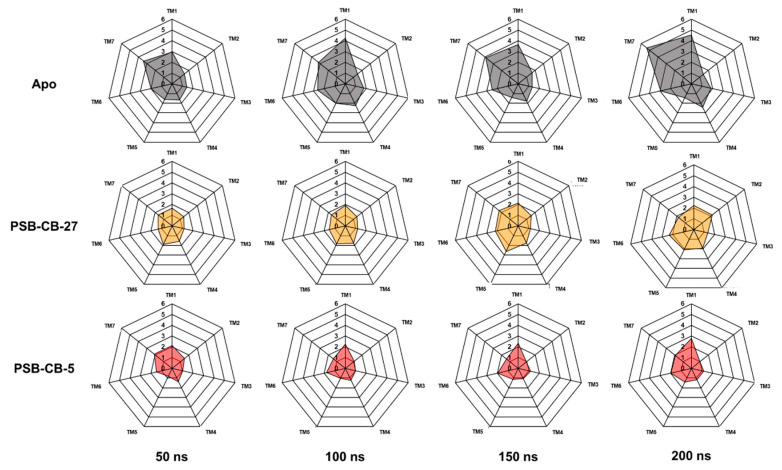
RMSD values for the transmembrane (TM) regions during the 200 ns MD simulation runs. Values were calculated based on the initial complex state after equilibration (0 ns).

To further investigate conformational changes in the receptor, RMSD values for each transmembrane-spanning helix were calculated (see Figure 5). Using the OPM database [46] seven transmembrane region segments were determined: TM1 (Ile23^1.33^–Ser48^1.58^), TM2 (Ile59^2.41^–Phe80^2.62^), TM3 (Glu91^3.22^–Ala117^3.53^), TM4 (Val139^4.43^–Tyr160^4.64^), TM5 (Ala183^5.34^–Val209^5.60^), TM6 (Ile231^6.34^–Phe254^6.57^) and TM7 (Trp267^7.34^–Val289^7.56^). The RMSD values amounted to 2.8, 1.0, 1.2, 1.2, 1.4, 2.0 and 1.9 Å for TM1–TM7, respectively, when comparing the TM regions of the complex of GPR18 and compound **4** at 0 ns and at 200 ns. For the complex with the larger antagonist **4**, the RMSD values amounted to 2.2, 2.2, 1.4, 1.9, 2.1, 2.3 and 1.8 Å for TM1–TM7, respectively. The higher RMSD values for antagonist **4** can be explained by the size of the compound when compared to **5**: the larger linker requires adaptation of the receptor, resulting in higher RMSD values. In contrast to the behavior of the antagonist-bound complexes, even higher RMSD values were observed for the unbound apo form of GPR18. Here, RMSD values of 4.6, 1.4, 1.8, 2.4, 1.4, 2.9 and 5.4 Å were calculated for TM1–TM7, respectively. Furthermore, the stabilization of TM1, TM6 and TM7 in the presence of an antagonist supports the theory of stabilization of an inactive state of the receptor upon binding of an antagonist [74,75,76].

Potential salt bridges within the receptor were analyzed to further investigate the mode of inhibition. Arg119^3.50^ of the DRY motif had been proposed to be located in an “arginine cage,” where it forms an ionic lock with Asp118^3.49^, thus stabilizing the inactive GPR18 [54,77]. Disruption of the ionic lock was postulated to contribute to receptor activation through facilitated movements of TM3 and TM6, resulting in conformational changes towards the intracellular lumen [54]. The authors concluded that stable salt bridges or H-bonds induce a rotamer of Arg119^3.50^, which is no longer present during receptor activation. The ionic lock between Asp118^3.49^ and Arg119^3.50^ was observed in the apo form of GPR18 during our 200 ns MD simulation run, which is consistent with previous studies [54]. Interestingly, we observed differences in the behavior of Arg119^3.50^ in the apo form as compared to the antagonist-bound complexes: in the apo form, the salt bridge between Arg119^3.50^ and Asp118^3.49^ formed after approximately 75 ns and was stable until the end of the MD simulation, while no similar interaction was observed for the antagonist-bound complexes. Asp118^3.49^ formed a stable salt bridge with Lys133^ICL2^ in the complexes but not in the apo form. This lysine is neither conserved in the three homology model templates nor in the two CB receptor subtypes. Furthermore, we observed stable ionic interactions of Asp85^ECL1^ with Lys22^N-terminus^ and of Asp162^ECL2^ with Lys161^ECL2^ in the antagonist-bound structures, which were not present in the apo form. Interaction of Glu131^ICL2^ with Lys137^4.41^ was observed in all three structures. The salt bridge between Glu228^6.31^ and Arg232^6.35^ was stable in the receptor apo form, which was not the case for the antagonist-bound structures. The trajectory for the salt bridge distances is presented in Supplementary Information (Figure S4). We conclude that the binding of an antagonist stabilizes several salt bridges within GPR18, resulting in the stabilization of an inactive conformation of the receptor.

**Figure 6:**
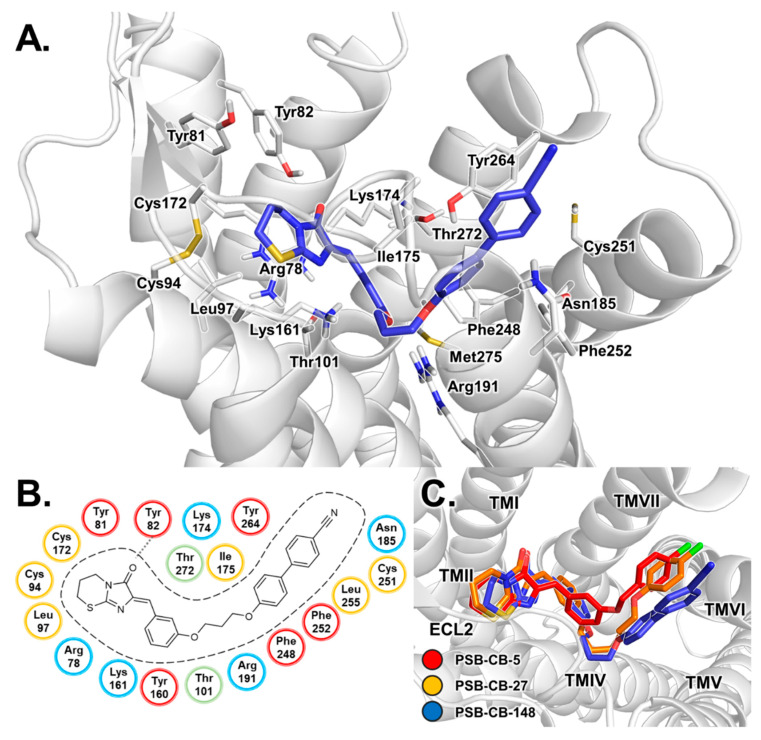
Proposed binding mode of antagonist **6**. (**A**) Docked pose of **6** in complex with the homology model of human GPR18 shown with the residues forming the binding pocket. (**B**) Schematic 2D representation of the binding pocket. For color code, see Figure 2. (**C**) Overlay of the proposed binding modes of GPR18 antagonists. Antagonist **4** is colored in orange, antagonist **5** in red, antagonist **6** in blue.

We additionally investigated the binding mode of a new potent antagonist, an analog of **4** and **5**, which has a more rigid substituent in position 4 of the phenyl group (a biphenyl derivative). Compound PSB-CB-148 (**6**) contains a *p*-cyano-biphenyl group which is larger and at the same time less flexible than the corresponding substituents in antagonists **4** and **5**. The imidazothiazinone group is predicted to bind in the same binding cavity as for compounds **4** and **5** (see Figure 6). The trajectory of the linker in the docked structure closely resembles the binding mode of compound **4**. Furthermore, the proximity of Arg191^5.42^ to both oxygen atoms in the linker indicates bidental H-bond interactions. The biphenyl moiety likely binds in a lipophilic binding cavity, where π–π interactions between the phenyl groups and the aromatic residues Phe248^6.51^, Phe252^6.55^ and Tyr264^7.31^ are feasible. Interactions of the terminal phenyl group with Cys251^6.54^ are not observed for **6**. Due to its decreased flexibility, the terminal group does not allow this interaction. The shift in the phenyl group is predicted to place the cyano moiety in close proximity to Asn185^5.39^. Upon inspection of Asn185^5.39^, several rotamers were found which could form H-bonds with the nitrile (see Figure S5 in Supplementary Information).

**Figure 7:**
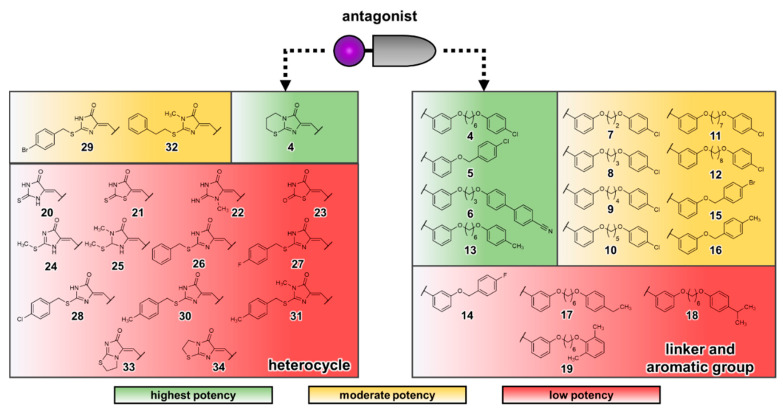
Schematic representation of the structure–activity relationships (SARs) of GPR18 antagonists. The different heterocycles shown contain a 4-chlorophenoxy group, while the compounds with varying aryl substituents and linker lengths contain the imidazothiazinone heterocycle. Compounds were categorized into three groups: highest potency (IC_50_ < 1 µM), moderate potency (1 µM < IC_50_ < 10 µM) and low potency (IC_50_ > 10 µM) based on their antagonistic activity.

The obtained data of the docking studies were used to re-analyze the SARs of previously published antagonists [21]. The summarized results are presented in Figure 7 (for structures, see Figures S6 and S7 in Supporting Information). The linker size was found to have an impact on the potency of the tested antagonists. The antagonist containing a hexyloxy linker (**4**) showed an almost 10-fold increase in potency compared to the analog with the shorter ethyloxy linker (**7**) (IC_50_ of 0.650 µM versus 5.00 µM). Prolongation of the ethyloxy linker resulted in increased inhibitory potency, with hexyloxy being optimal (IC_50_ = 0.650 µM), while larger linkers, i.e., heptyloxy (**11**) and octyloxy (**12**), led to slightly less potent antagonists (IC_50_ = 1.71 and 1.15 µM). Our docking results suggest that the hexyloxy linker is required for the 4-chlorophenoxy moiety to reach the aromatic binding pocket and to form hydrophobic interactions with Cys251^6.54^. The shorter alkyloxy linker is less well stabilized in the hydrophobic cavity formed by Tyr160^4.64^, Ile175^ECL2^, Phe248^6.51^ and Met275^7.42^. The decrease in potency observed for compounds **11** and **12** despite their higher lipophilicity could be explained by limited space in the binding cavity or unfavorable adaptation of the alkyloxy linker, resulting in a shifted binding position for the 4-chlorophenoxy moiety which prohibits optimal interaction with Cys251^6.54^. Among the smaller compounds missing an additional linker between the benzylidene and the substituted phenoxy ring, the most potent antagonists contained a hydrophobic substituent in position 4 of the phenyl ring (compounds **5**, **14**–**16**). Hydrophobic interactions of substituents in position 4 of the phenoxy residue with Cys251^6.54^ are supported by acceptance of both chlorine and methyl groups in compounds **4** and **13,** resulting in comparable IC_50_ values (0.650 and 0.238 µM). The potency (IC_50_ values) of the compounds decreased in the following rank order Cl (0.279 µM) > Br (1.73 µM) ≥ CH_3_ (3.59 µM) > F (> 10 µM), indicating that the size and lipophilicity of the substituent plays a major role. Decreased potency observed for antagonists containing larger substituents in position 4 such as ethyl (**17**) or isopropyl (**18**) can be explained by the limited space of the binding pocket in proximity to Cys251^6.54^. Moreover, the substitution position on the phenyl ring proved to have an effect on the potency of the compounds. Antagonist **19** (*o*,*o*-dimethyl-substituted), for example, was inactive (IC_50_ > 10 µM). Antagonists containing different heterocycles in place of the imidazothiazinone moiety (**20**–**34**) showed lower potency as compared to antagonist **5**. In our homology model, two aromatic residues close to the hydrophobic binding pocket, Tyr81^2.63^ and Trp87^ECL1^, may form π–π interactions with antagonists containing an additional aromatic group attached to the heterocycle (see Figure S8 in Supporting Information). The ethylthio linker connecting the imidazolone ring with the phenyl ring in compound **32** might be beneficial to enable proper binding for π–π interactions. The results suggest that the imidazothiazinone heterocycle is optimal to allow hydrophobic packing in the binding pocket close to the disulfide bridge of ECL2.

In conclusion, the docking studies, MD simulations and SARs of imidazothiazinones as well as antagonists containing smaller heterocycles further support our suggested binding mode of an aromatic and lipophilic binding pocket of the human GPR18 for antagonists. The most potent antagonists of this series likely interact with Cys251^6.54^ through lipophilic interactions, and this additional interaction is predicted to be the reason for their high potency.

### 3.3. Binding Mode of THC

As a next step, we explored the most likely binding pocket for the GPR18 agonist THC (**1**). The ability of the potent CB receptor agonist THC to activate GPR18 with moderate potency had led to the suggestion to classify GPR18 as a novel CB receptor subtype [19]. Lipophilicity is a feature shared by GPR18 agonists and antagonists [78,79]. THC is regarded as a promiscuous ligand acting not only at cannabinoid but also at several non-cannabinoid receptors [80,81,82,83,84,85]. Studies on the binding mode of cannabinoids at the cannabinoid receptors CB_1_ and CB_2_ proposed a binding portal between TM6 and TM7 from the lipid-facing side of the receptor for the entrance of agonists [86,87,88]. Such entry is unique among GPCRs, as ligands typically reach the binding pocket between TM3 and TM7 from the extracellular lumen.

To date, two crystal structures of the CB_1_ receptor bound to THC-related compounds are available (PDB-ID: 5XR8, 5XRA) [38]. As observed for many other GPCRs, the agonist binding site, which is very lipophilic in the case of the CB_1_ receptor, is located between a highly conserved Trp^6.48^ and ECL2 [89,90]. The tricyclic THC ring system is stabilized through lipophilic as well as π–π interactions with an aromatic cluster (Phe170^2.57^, Phe174^2.61^, Phe177^2.64^, Phe189^3.25^, Phe268^ECL2^, Phe379^7.35^). Several previous mutagenesis studies have confirmed the key role of the aromatic residues for the binding of cannabinoids [38,91,92,93]. The alkyl chain of the agonists extends towards a binding cleft formed by several lipophilic residues (Leu193^3.29^, Val196^3.32^, Tyr275^5.39^, Leu276^5.40^, Leu359^6.51^ and Met363^6.55^).

Given the low sequence similarity between the cannabinoid receptors CB_1_, CB_2_, and GPR18 (18.7 and 23.7%, respectively), similar binding of the THC ring system in GPR18 cannot be taken for granted. Amino acid residues Val196^3.32^, Phe268^ECL2^, Tyr275^5.39^, Met363^6.55^ and Phe379^7.35^ are conserved in both CB receptor subtypes but replaced in GPR18 by leucine, serine, arginine, phenylalanine and glycine, respectively (see Figure S9 in Supplementary Information for multiple sequence alignment). Phe174^2.61^ and Leu193^3.29^, but not Leu359^6.51^, are conserved among all three receptors. The absence of the aromatic network responsible for the binding of the THC ring system in the CB receptors suggests a different binding mode for the agonist THC at GPR18.

**Figure 8:**
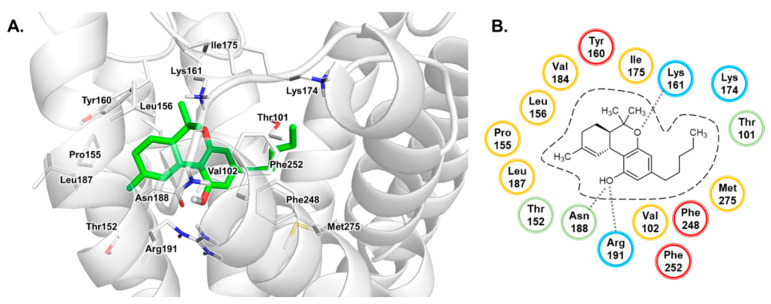
Proposed binding mode of ∆^9^-tetrahydrocannabinol (THC) in the homology model of human GPR18. (**A**) The receptor is displayed in cartoon representation, the amino acid residues (white) and THC (**1**, green) are shown as stick models. Oxygen atoms are colored in red, nitrogen atoms in blue, sulfur atoms in yellow. (**B**) Schematic 2D representation of the binding pocket. For color code, see Figure 2.

Docking studies of THC were performed using the generated homology model of the human GPR18. We observed that THC appears to bind closer to TM4 and TM5 as compared to the cannabinoids in the X-ray crystal structures of the CB_1_ receptor (see Figure 8). The phenyl group of the tricyclic THC ring system is predicted to bind in a cleft formed by several lipophilic (Val102^3.33^, Ile175^ECL2^, Phe248^6.51^, Phe252^6.55^) and hydrophilic (Lys161^ECL2^, Lys174^ECL2^, Asn188^5.39^, Arg191^5.42^, His249^6.52^) amino acid residues. H-bond interactions are feasible for the oxygen atoms of the chromene moiety and Lys161^ECL2^, as well as the hydroxy group and Asn188^5.39^ and Arg191^5.42^. The cyclohexenyl moiety is likely accommodated in a lipophilic binding pocket formed by Thr152^4.56^, Pro155^4.59^, Leu156^4.60^, Val184^5.35^ and the alkyl side chain of Arg191^5.42^. The alkyl group of the agonist likely projects towards TM7, where it can be stabilized through lipophilic interactions with Phe248^6.51^, Phe252^6.55^ and Met275^7.42^. The binding modes of THC in the CB_1_ receptor as compared to GPR18 are shown in Figure S10 of Supporting Information.

We propose that the tricyclic THC ring system binds in a binding cavity of GPR18 distant to the orthosteric binding site of the CB_1_ receptor. The absence of aromatic residues in ECL2 of GPR18 may contribute to the proposed shifted binding mode of THC, as π–π stacking with a phenylalanine in position 2.57 is not possible. However, the binding cleft for the alkyl chain is predicted to be overlapping in both receptors. It should be pointed out that THC displays much higher potency at CB_1_ (and CB_2_) receptors as compared to GPR18.

**Figure 9:**
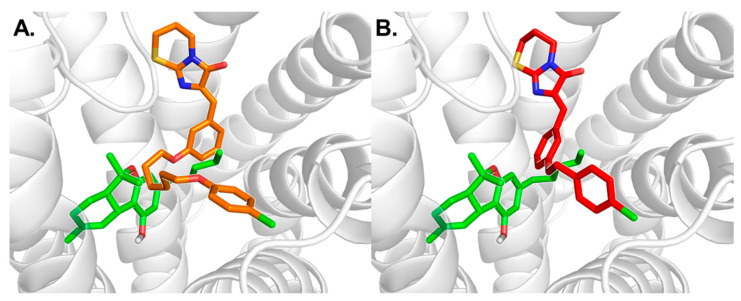
(**A**) Comparison of the proposed binding mode of THC (green) and antagonist **4** (orange) at human GPR18. (**B**) Comparison of the proposed binding modes of THC and antagonist **5** (red).

Our results suggest that THC shares a common binding pocket with the imidazothiazinone antagonists (see Figure 9). While the imidazothiazinone moiety of the antagonists is predicted to bind in a lipophilic pocket formed by amino acid residues of TM2 and TM7, the benzylidene group is suggested to project towards the putative binding site of the chromene and alkyl group of THC. This is supported by experimental data showing that imidazothiazinone antagonists containing lipophilic residues act as competitive antagonists versus THC [21].

## 4. Conclusions

Since only approximately 10% of the non-olfactory GPCRs are covered by structural studies, meaningful prediction of ligand-binding modes represents one of the greatest challenges in molecular modeling [94]. In particular, homology modeling assessment of receptors with no resolved closely related crystal structures requires further experimental validation. In the present study, we generated a homology model of the orphan GPR18 and predicted the binding modes of the confirmed agonist THC as well as the most potent class of antagonists containing an imidazothiazinone scaffold. Despite the lack of closely related X-ray crystal structures, we successfully performed docking and MD simulation studies of antagonist complexes which were in agreement with the extensive published SAR data. The investigated potent antagonists are predicted to share the same binding site for the imidazothiazinone core. The linker of the antagonists is likely accommodated in a lipophilic binding cleft shared by the alkyl chain of the agonist THC. The 200 ns MD simulation runs suggested stabilization of a receptor conformation by antagonists which was not observed for the unbound receptor structure. Stabilization of a salt bridge between Asp118^3.49^ and Lys133^ICL2^ through imidothiazinone-based antagonists may play a role in the inhibition mechanism. Our docking studies suggest a different binding mode of the agonist THC in GPR18 as compared to that observed in cannabinoid receptors. However, future structural studies will be required to confirm the proposed interactions. The presented data provide a well-founded hypothesis that will support the rational design of new ligands for this poorly investigated receptor which has potential as a future drug target.

## Supplementary Materials

The following are available online at https://www.mdpi.com/2218-273X/10/5/686/s1, Figure S1: Multiple sequence alignment of GPR18 for homology modeling, Figure S2: Time scale of the molecular dynamics simulation of antagonist 4, Figure S3: Time scale of the molecular dynamics simulation of antagonist 5, Figure S4: Trajectories of salt bridges, Figure S5: Possible interaction of antagonist 6 with Asn185, Figure S6: Structures of GPR18 imidazothiazinone antagonists, Figure S7: Structures of GPR18 antagonists with modification of the core structure, Figure S8: Comparison of the binding modes of antagonists 5 and 32, Figure S9: Multiple sequence alignment of human GPR18 and the cannabinoid receptors CB1 and CB2, Figure S10: Comparison of the binding mode of THC to GPR18 with the binding of THC derivatives to the CB1 receptor, Figure S11: Multiple Sequence alignment of top 10 scoring BLAST sequences, Figure S12: Residues considered to be important for the binding of antagonists, Table S1: Results for 10 scoring BLAST sequences for GPR18.
