# Supplementary material for: Computational Investigations on the Binding Mode of Ligands for the Cannabinoid-Activated G Protein-Coupled Receptor GPR18
Source: Biomolecules. 2020 Apr 29;10(5):686. doi: 10.3390/biom10050686 (PMC7277601; doi:10.3390/biom10050686)
Supplement: Supplementary file 1 [file biomolecules-10-00686-s001.pdf]

Supporting Information

Computational Investigations on the Binding Mode of Ligands for the Cannabinoid-Activated G Protein-Coupled Receptor GPR18

Alexander Neumann,[1,2] Viktor Engel,[1] Andhika B. Mahardhika,[1,2] Clara T. Schoeder,[1,2] Vigneshwaran Namasivayam,[1] Katarzyna Kieć-Kononowicz,[3] and Christa E. Müller[1,2*]

[1] PharmaCenter Bonn, Pharmaceutical Institute, Pharmaceutical Sciences Bonn (PSB), Pharmaceutical & Medicinal Chemistry, University of Bonn, Bonn 53121, Germany

[2] Research Training Group 1873, University of Bonn, Bonn 53127, Germany

[3] Department of Technology and Biotechnology of Drugs, Faculty of Pharmacy, Jagiellonian University Medical College, Medyczna 9, 30 - 688 Krakow, Poland.

* Correspondence: christa.mueller@uni-bonn.del; Tel.: +49-228-73-2301. Fax: +49-228-73-2567

Table of Contents

Figure S1. Multiple sequence alignment of GPR18 for homology modeling 2

Figure S2. Time scale of the molecular dynamics simulation of antagonist **4** 3

Figure S3. Time scale of the molecular dynamics simulation of antagonist **5** 4

Figure S4. Trajectories of salt bridges 5

Figure S5. Possible interaction of antagonist **6** with Asn185 6

Figure S6. Structures of GPR18 imidazothiazinone antagonists 7

Figure S7. Structures of GPR18 antagonists with modification of the core structure 8

Figure S8. Comparison of the binding modes of antagonists **5** and **32** 9

Figure S9. Multiple sequence alignment of human GPR18 and the cannabinoid receptors CB_1 and CB_2 10

Figure S10. Comparison of the binding mode of THC to GPR18 with the binding of THC derivatives to the CB_1 receptor 11

References 12

```
4xnv        1 TGFQF-YYLPAVYILVFIIGFLGNSVAIWMFVFHMKPWSGISVYMFNLALADFLYVLTLP   59
5c1m        1 PSMVTAITIMALYSIVCVVGLFGNFLVMYVIVRYTKMKTATNIYIFNLALADALATSTLP   60
5xsz        1 DNFKYPLYSM-VFSIVFMVGLITNVAAMYIFMCSLKLRNETTTYMMNLVVSDLLFVLTLP   59
GPR18       1 --DEYKIAALVFYSCIFIIGLFVNITALWVFSCTTKKRTTVTIYMMNVALVDLIFIMTLP   58

4xnv       60 ALIFYYFNKTDWIFGDAMCKLQRFIFHVNLYGSILFLTCISAHRYSGVVYP-KSLGRLKK  118
5c1m       61 FQSVNYLMGT-WPFGNILCKIVISIDYYNMFTSIFTLCTMSVDRYIAVCHPVKALDFRTP  119
5xsz       60 LRVFYFVQQN-WPFGSLLCKLSVSLFYTNMYGSILFLTCISVDRFLAIVYPFRSRGLRTK  118
GPR18      59 FRMFYYAKDE-WPFGEYFCQILGALTVFYPSIALWLLAFISADRYMAIVQPKYAKELKNT  117

4xnv      119 KNAICISVLVWLIVVVAISPILFY-SGTGVRKNKTITCYDTTSDEYLRSYFIYSMCTTV-  176
5c1m      120 RNAKIVNVCNWILSSAIGLPVMFM--ATTKYRQGSIDCTLTFSHPTWYWENLLKICVFI-  176
5xsz      119 RNAKIVCAAVWVLVLSGSLPTGFMLNSTNKLENNSISCF------EWK-SHLSKVVIFIE  171
GPR18     118 CKAVLACVGVWIMTLTTTTPLLLLYKDPDK-DSTPATCLKISDIIYLKAVNVLNLTRLT-  175

4xnv      177 -AMFCVPLVLILGCYGLIVRALIYKEPL---------RRKSIYLVIIVLTVFAVSYIPFH  226
5c1m      177 -FAFIMPVLIITVCYGLMILRLKSVRMLSGSKEKDRNLRRITRMVLVVVAVFIVCWTPIH  235
5xsz      172 TVGFLIPLMLNVVCSAMVLQTLRRPNTVL-------NKKKILRMIIVHLFIFCFCFIPYN  224
GPR18     176 -FFFLIPLFIMIGCYLVIIHNLLHGRTSK---LKPKVKEKSIRIIITLLVQVLVCFMPFH  231

4xnv      227 VMKTMNLRARLDFQTPAMCAFNDRVYAT-YQVTRGLASLNSCVNPILYFLAGDTFRRR  283
5c1m      236 I--YVIIKALI---TIP----ETTFQTVSWHFCIALGYTNSCLNPVLYAFLDENFKRC  284
5xsz      225 V--NLVFYSLVRTNTLKGCAAESVVRTI-YPIALCIAVSNCCFDPIVYYFTSETIQNS  279
GPR18     232 I--CFAFLMLGT---------GENSYNPWGAFTTFLMNLSTCLDVILYYIVSKQFQAR  278
```

Figure S1. Multiple sequence alignment of the human GPR18 and the templates chosen for homology modeling.

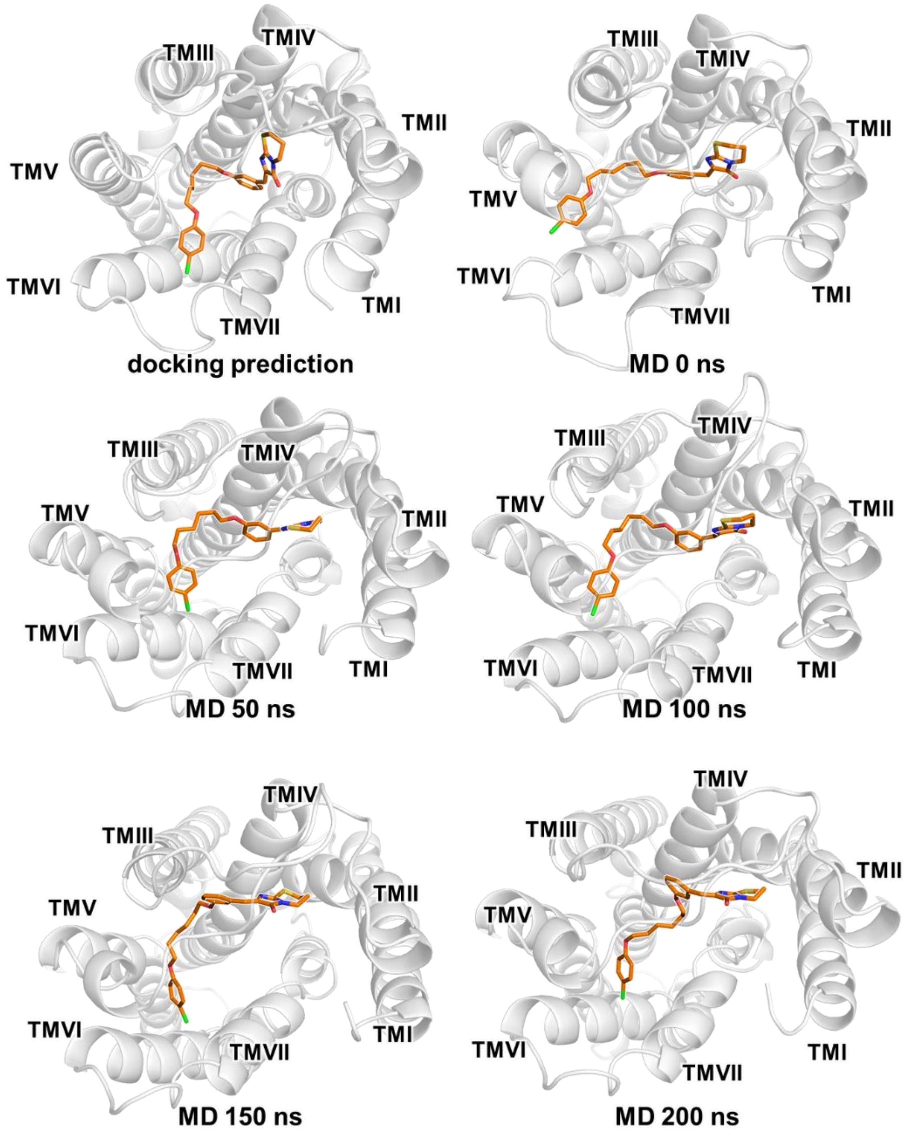

Figure S2. Time scale of the molecular dynamics (denoted 'MD') simulation of GPR18 homology model complex with antagonist **4**. The docking prediction which was used for the simulation run is shown at the top left corner. 0 ns presents the complex after relaxation steps.

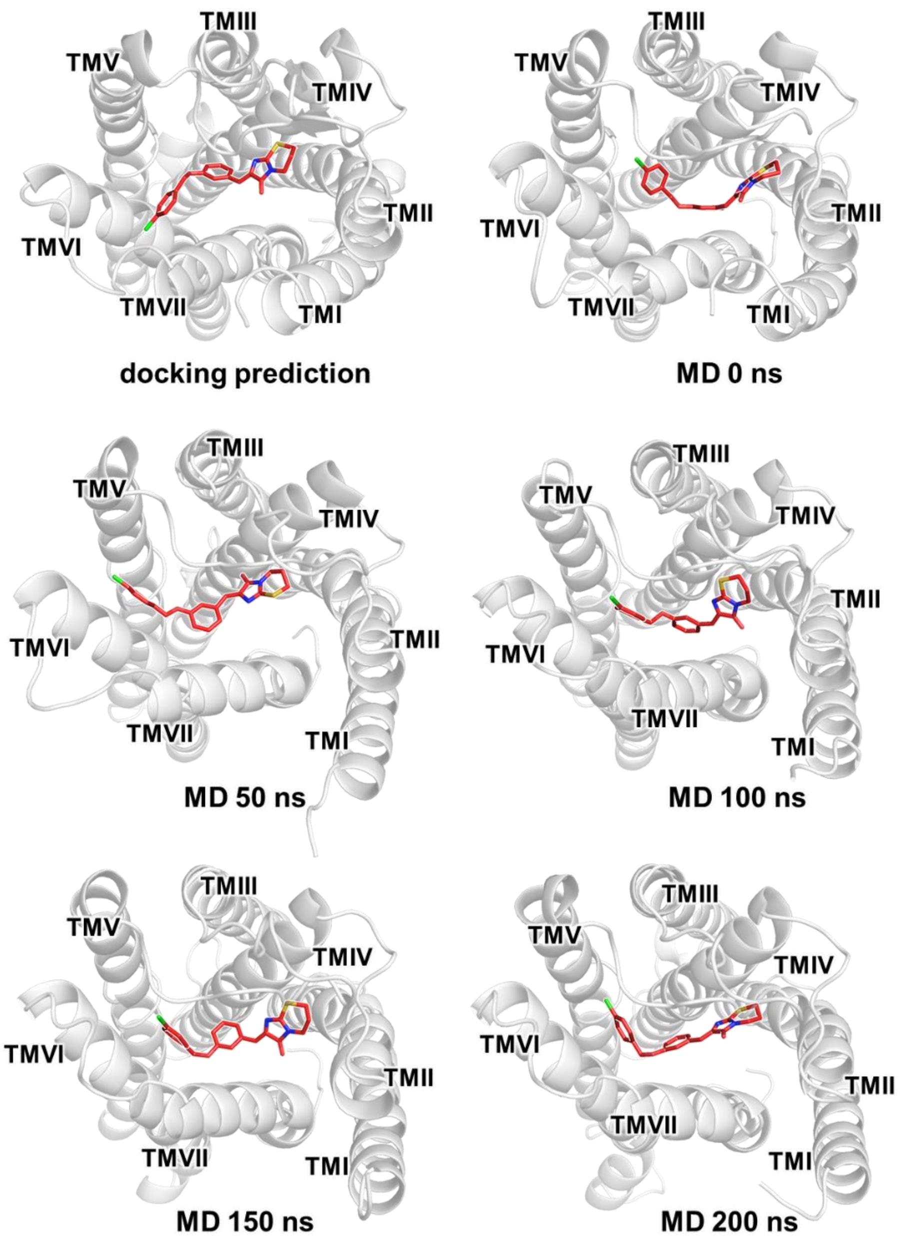

Figure S3. Time scale of the molecular dynamics (denoted 'MD') simulation of GPR18 homology model complex with antagonist **5**. The docking prediction which was used for the simulation run is shown at the top left corner. 0 ns presents the complex after relaxation steps.

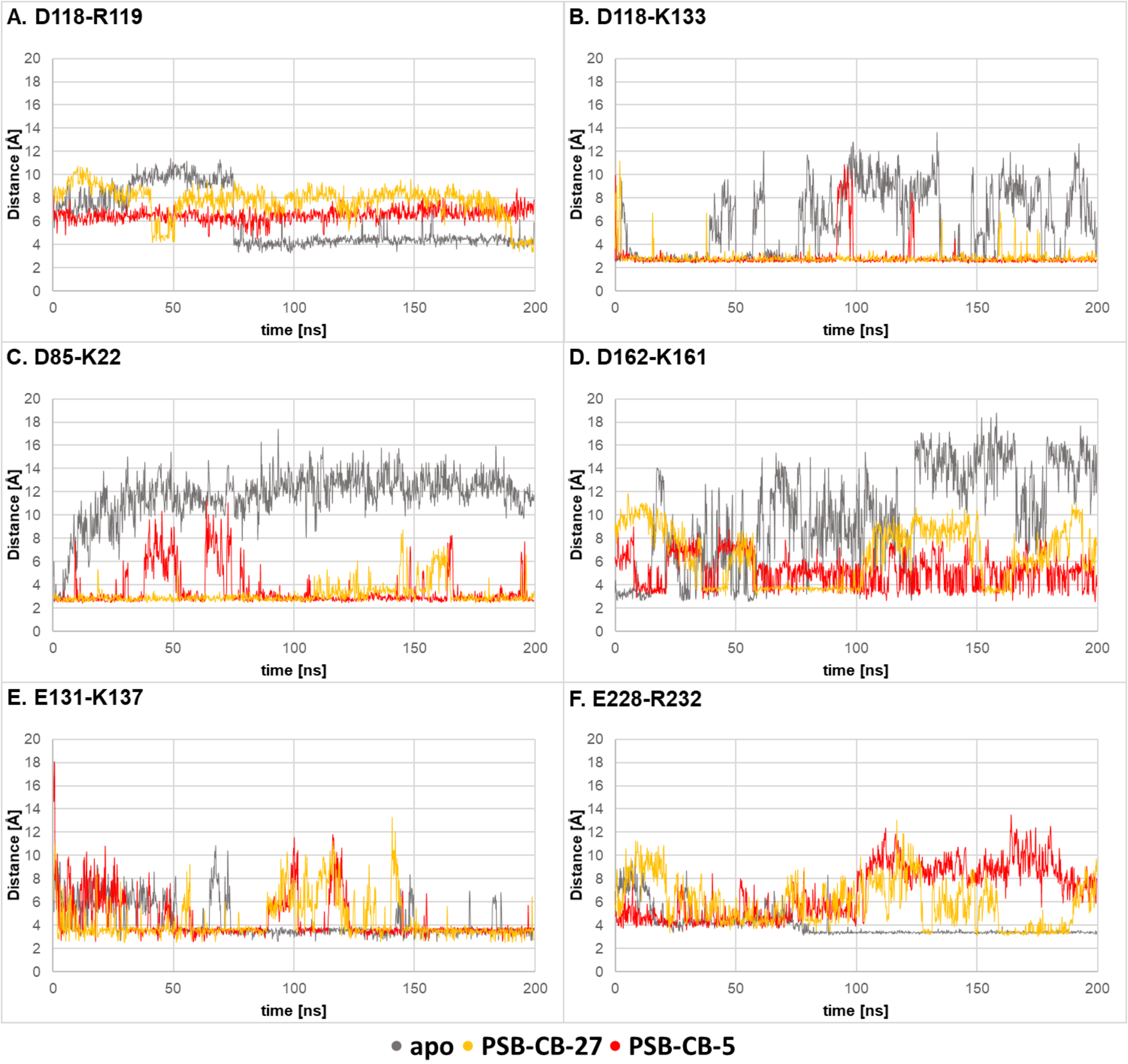

Figure S4. Trajectories of salt bridges during the 200 ns MD simulation runs.

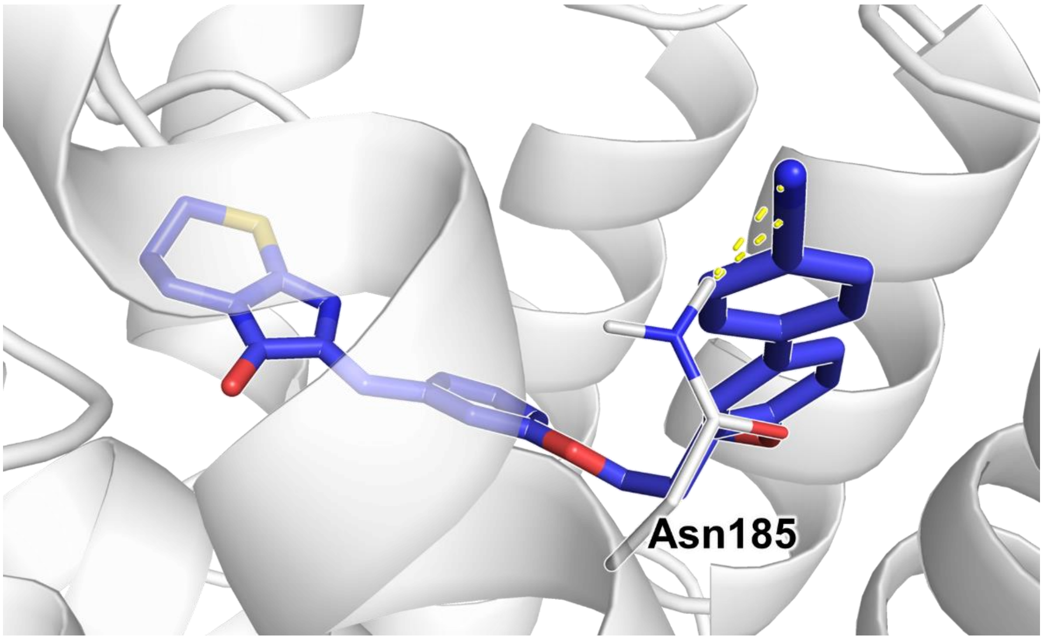

Figure S5. Possible interaction of antagonist **6** with a rotamer of Asn185.

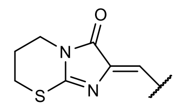

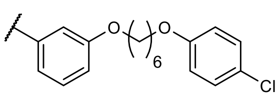

4 PSB-CB-27
(0.650 µM)

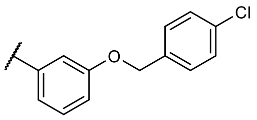

5 PSB-CB-5
(0.279 µM)

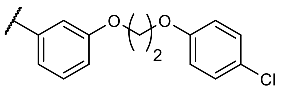

7 CB-30
(5.00 µM)

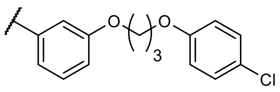

8 CB-25
(3.25 µM)

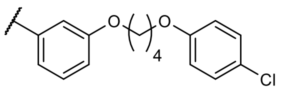

9 CB-28
(2.38 µM)

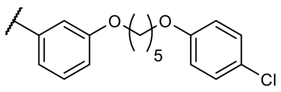

10 CB-26
(1.14 µM)

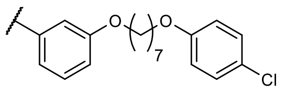

11 CB-101
(1.71 µM)

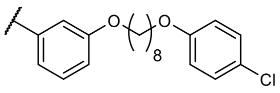

12 CB-29
(1.15 µM)

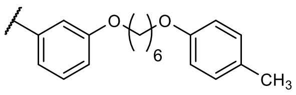

13 CB-92
(0.238 µM)

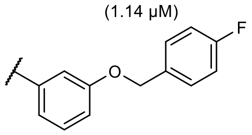

14 CB-21
(>10% µM (3%))

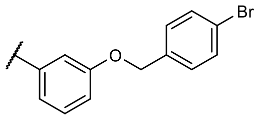

15 CB-20
(1.73 µM)

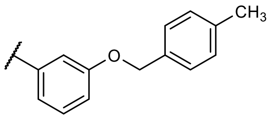

16 PSB CB-22
(3.59 µM)

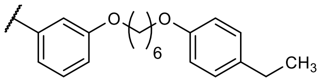

17 CB-94
(25.0 µM)

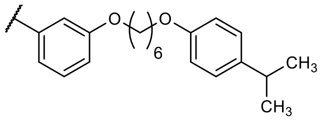

18 CB-98
(>10 µM (24%))

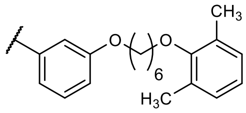

19 CB-96
(ca. 10 µM)

Figure S6. Structures of GPR18 imidazothiazinone antagonists with their respective IC_{50} values in brackets. For IC_{50} values > 10 µM the percent inhibition of agonist-induced luminescence signal at 10 µM is given. Biological results were taken from published studies [1].

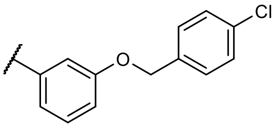

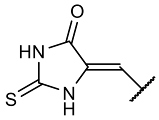

20 CB-59
(33.0 µM)

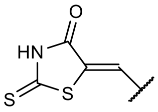

21 CB-61
(>10 µM (22%))

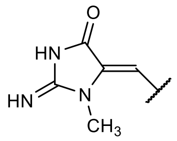

22 CB-62
(>10 µM (4%))

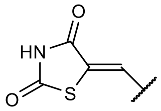

23 CB-63
(>10 µM (-10%))

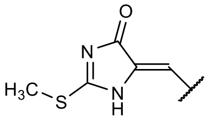

24 CB-60
(13.8 µM)

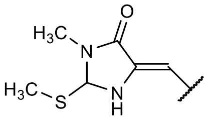

25 CB-66
(>10 µM (23%))

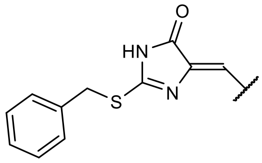

26 CB-70
(>10 µM (3%))

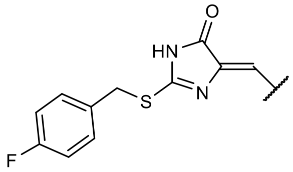

27 CB-74
(>10 µM (27%))

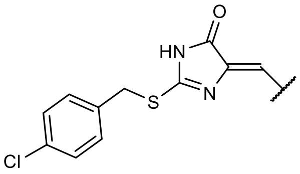

28 CB-76
(>10 µM (35%))

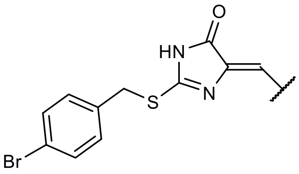

29 CB-71
(2.29 µM)

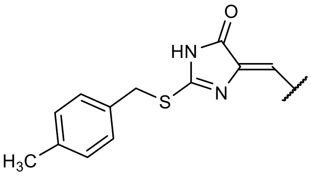

30 CB-75
(>10 µM (0%))

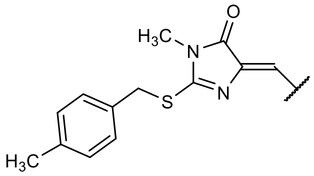

31 CB-77
(>10 µM (17%))

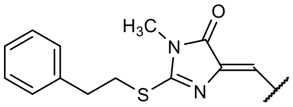

32 CB-78
(4.78 µM)

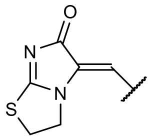

33 CB-72-1,2
(>10 µM (25%))

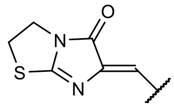

34 CB-72-2,3
(>10 µM (40%))

Figure S7. Structures of GPR18 antagonists with modification of the core structure with their respective IC_{50} values in brackets. For IC_{50} values > 10 µM the percent inhibition of agonist-induced luminescence signal at 10 µM is given. Biological results were taken from published studies [1].

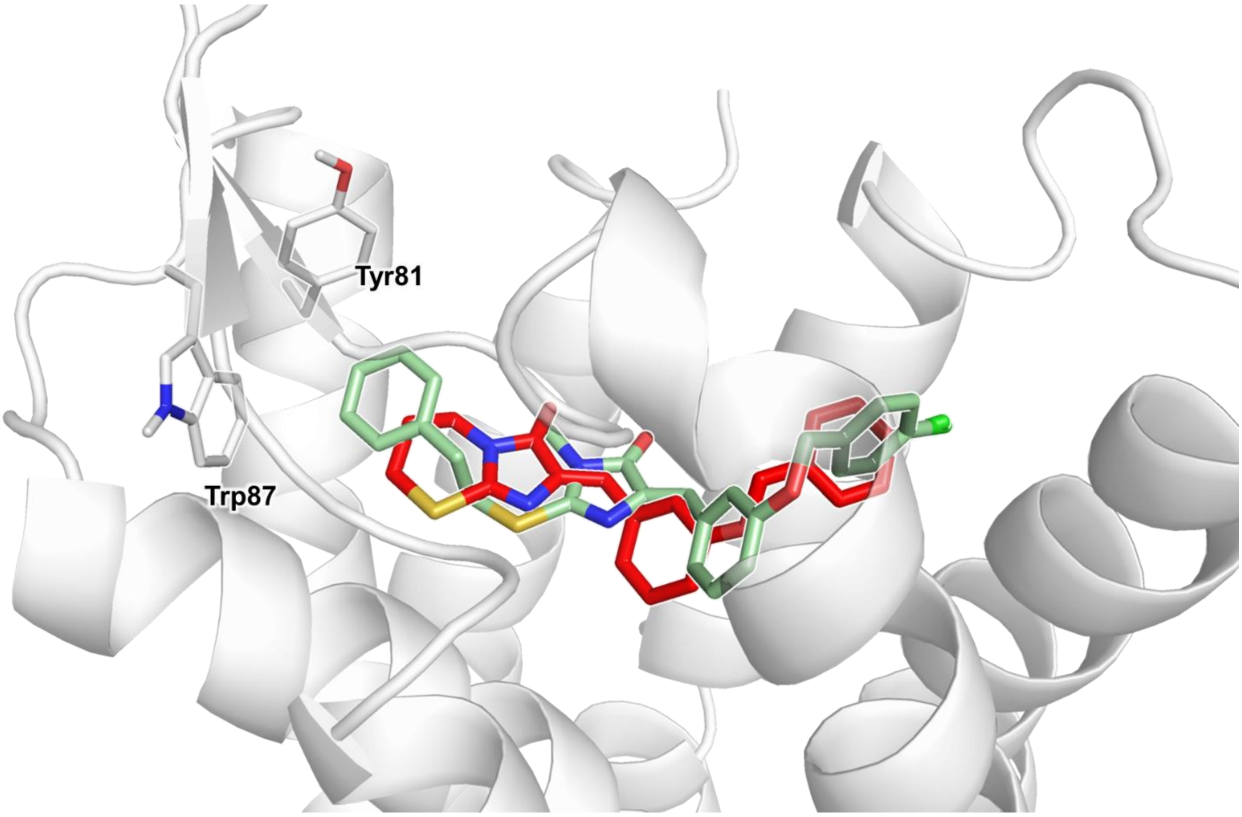

Figure S8. Comparison of the putative binding mode of antagonist **32** (green) and predicted binding mode of antagonist **5**.

```
sp|Q14330|GPR18_HUMAN      ------------------------------------------------------------     0
sp|P21554|CNR1_HUMAN       MKSILDGLADTTFRTITTDLLYVGSNDIQYEDIKGDMASKLGYFPQKFPLTSFRGSPFQE     60
sp|P34972|CNR2_HUMAN       ------------------------------------------------------------     0

                                                                                   TMI
sp|Q14330|GPR18_HUMAN      MITLNNQDQPVPFNS------------------------------------SHPDEYKIA     24
sp|P21554|CNR1_HUMAN       KMTAGDNPQLVPADQVNITEFYNKSLSSFKENEENIQCGENFMDIECFMVLNPSQQLAIA     120
sp|P34972|CNR2_HUMAN       -----------------MEECWVT------EIANGSKDGLDSNPMKDYMILSGPQKTAVA37
                                                                            .   ::   :*
                                                                       TMII
sp|Q14330|GPR18_HUMAN      ALVFYSCIFIIGLFVNIT---ALWVFSCTTK-KRTTVTIYMMNVALVDLI----FIMT-L     75
sp|P21554|CNR1_HUMAN       VL-----SLTLGTFTVLENLLVLCVILHSRSLRCRPSYHFIGSLAVADLLGSVIFVYSFI     175
sp|P34972|CNR2_HUMAN       VL-----CTLLGLLSALENVAVLYLILSSHQLRRKPSYLFIGSLAGADFLASVVFACSFV     92
                           .*         :* :  :     .* ::  :  .  :      ::  .:* .*::    *   : :
                                          TMIII
sp|Q14330|GPR18_HUMAN      PFRMFYYAKDEWPFGEYFCQILGALT-VFYPSIALWLLAFISADRYMAIVQPKYAKELKN     134
sp|P21554|CNR1_HUMAN       DFHVFHR-KDS---RNVFLFKLGGVTASFTASVGS--LFLTAIDRYISIHRPLAYKRIVT     229
sp|P34972|CNR2_HUMAN       NFHVFHG-VDS---KAVFLLKIGSVTMTFTASVGS--LLLTAIDRYLCLRYPPSYKALLT     146
                            *::*:   *.       *    :*.:*  *  *:.   * : : ***:.:  *   * : .
                              TMIV                                             TMV
sp|Q14330|GPR18_HUMAN      TCKAVLACVGVWIMTLTTTTPLLLLYKDPDKDSTPATCLKISDIIYLKAVNVLNLTRLTF     194
sp|P21554|CNR1_HUMAN       RPKAVVAFCLMWTIAIVIAVLPLLGWNCEK---LQSVCSDIFPHI--------DETYLMF     278
sp|P34972|CNR2_HUMAN       RGRALVTLGIMWVLSALVSYLPLMGWTCCP-----RPCSELFPLI--------PNDYLLS     193
                             :*:::   :* ::    :    *: :.           *  .:    *           *

sp|Q14330|GPR18_HUMAN      FFLIPLFIMIG---CYLV---------------------IIHNLLHGRTSKLKPKVKEKS     230
sp|P21554|CNR1_HUMAN       WIGVTSVLLLFIVYAYMYILWKAHSHAVRMIQRGTQKSIIIHTSEDGKVQVTRPDQARMD     338
sp|P34972|CNR2_HUMAN       WLLFIAFLFSGIIYTYGHVLWKAHQHVASLSGH-------------QDRQVPGMARMRLD     240
                           :: .   .::       *                                    .       . .
                           TMVI                                               TMVII
sp|Q14330|GPR18_HUMAN      IR---IIITLLVQVLVCFMPFHICFAFLMLGTGENSYNPWGAFTTFLMNLSTCLDVILYY     287
sp|P21554|CNR1_HUMAN       IRLAKTLVLILVVLIICWGPLLAIMVYDVFGKMNKLIKTVFAFCSMLCLLNSTVNPIIYA     398
sp|P34972|CNR2_HUMAN       VRLAKTLGLVLAVLLICWFPVLALMAHSLATTLSDQVKKAFAFCSMLCLINSMVNPVIYA     300
                           :*     :   :*. :::*: *.   :..  :   . ..   :   ** ::*   :.: :: ::*

sp|Q14330|GPR18_HUMAN      IVSKQFQARVISVMLYRNYLRSMRRKSFRSGSLRSLSNINSEML----------------     331
sp|P21554|CNR1_HUMAN       LRSKDLRHAFRSMFP--SC----------EGTAQPLDNSMGD----SDCLHKHANN----     438
sp|P34972|CNR2_HUMAN       LRSGEIRSSAHHCLA--HW----------KKCVRGLGSEAKEEAPRSSVTETEADGKITP     348
                           : * :::        :                   .    : *..    :

sp|Q14330|GPR18_HUMAN      -----------------------------------  331
sp|P21554|CNR1_HUMAN       -AASVHRAAESCIKSTVKIAKVTMSVSTDTSAEAL  472
sp|P34972|CNR2_HUMAN       WPDSRDLDLSDC-----------------------  360
```

Figure S9. Multiple sequence alignment of human GPR18 and the cannabinoid receptors CB_1 and CB_2. Residue positions involved in the binding of cannabinoid agonists in the X-ray crystal structure of CB_1 receptor are highlighted.

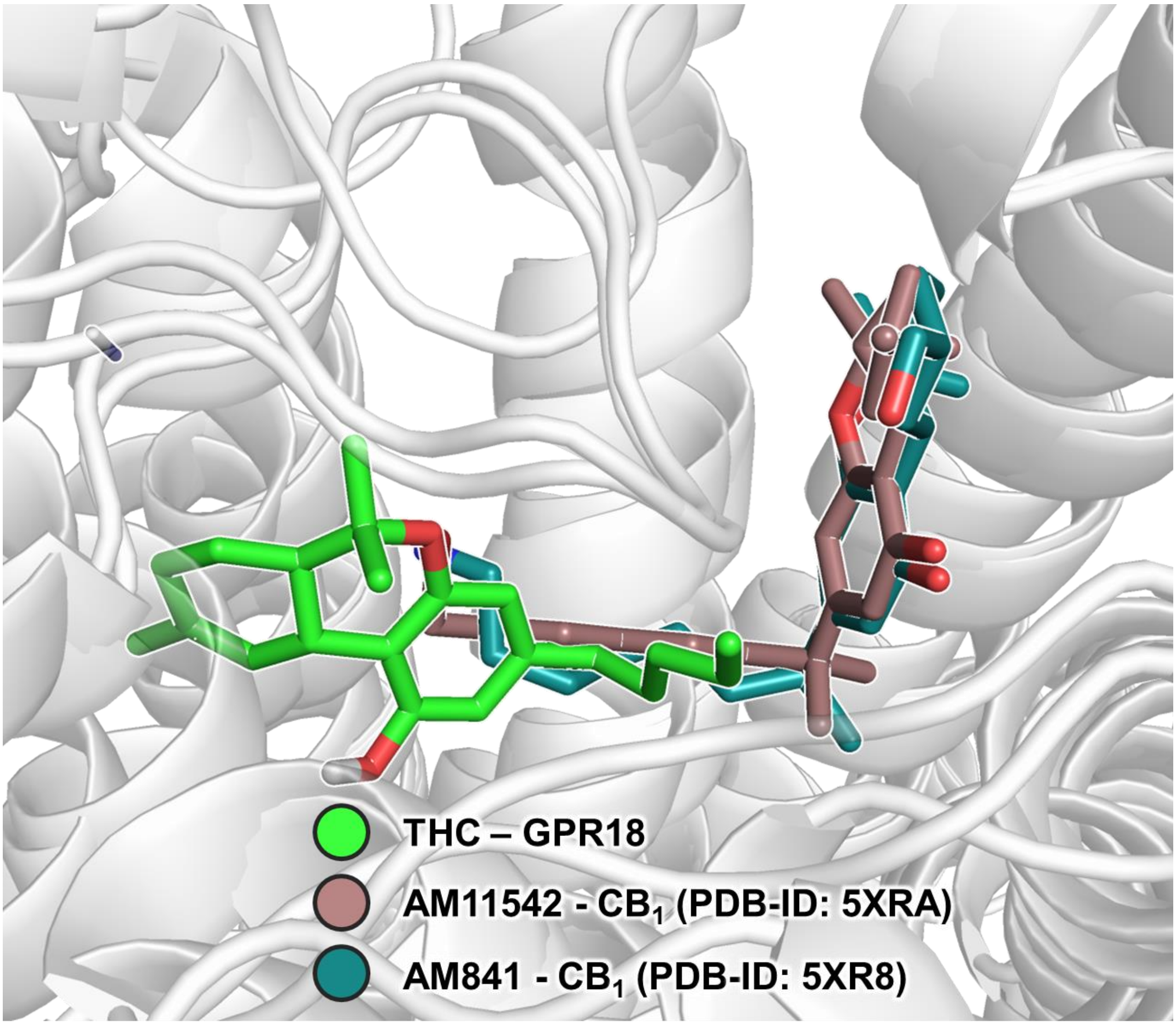

Figure S10. Comparison of the proposed binding mode of THC to GPR18 with the binding of THC derivatives to the CB_1 receptor as observed in the crystal structure [2].

References

1. Schoeder, C. T.; Kaleta, M.; Mahardhika, A. B.; Olejarz-Maciej, A.; Łażewska, D.; Kieć-Kononowicz, K.; Müller, C. E. Structure-activity relationships of imidazothiazinones and analogs as antagonists of the cannabinoid-activated orphan G protein-coupled receptor GPR18. *Eur. J. Med. Chem.* **2018**, *155*, 381–397.
2. Hua, T.; Vemuri, K.; Nikas, S. P.; Laprairie, R. B.; Wu, Y.; Qu, L.; Pu, M.; Korde, A.; Jiang, S.; Ho, J.-H.; *et al.* Crystal structures of agonist-bound human cannabinoid receptor CB1. *Nature* **2017**, *547*, 468–471.
